# When What You See Isn’t What You Get: How Environmental Cues Modify Longevity and Behavior

**DOI:** 10.1093/geroni/igaf122.967

**Published:** 2025-12-31

**Authors:** Scott Leiser

**Affiliations:** University of Michigan, Ann Arbor, Michigan, United States

## Abstract

Research in geroscience has identified interventions that can extend lifespan across model organisms. These interventions often involve exposure to mild environmental stressors that activate stress-response pathways to promote longevity. However, exposure to environmental stress can also change an animal’s behavior and mental state. Our research focuses on the environmental cues and signaling pathways that affect longevity cell non-autonomously, and how they feedback to regulate mental state or behavior. This presentation will focus on the dietary restriction (DR) induced nematode longevity gene, fmo-2, whose expression is regulated by food cues and whose activity modifies behavior. We find that multiple food cues repress fmo-2 induction in the DR state through well-conserved bioamine signaling pathways. We further find that loss of fmo-2 and overexpression of fmo-2 each lead animals to exhibit altered sensory perception and decision-making relative to wild-type (WT) controls. These behavioral changes likely represent metabolic feedback to the nervous system, and interventions that rescue behavior also rescue some, but not all of the other health and longevity phenotypes of the animals. These results establish a feedback loop for environmental response and demonstrate the importance of examining pleiotropic effects in promising longevity interventions.

